# Marine natural products and human immunity: novel biomedical resources for anti-infection of SARS-CoV-2 and related cardiovascular disease

**DOI:** 10.1007/s13659-024-00432-4

**Published:** 2024-01-29

**Authors:** Chunsong Hu

**Affiliations:** Department of Cardiovascular Medicine, Jiangxi Academy of Medical Science, Nanchang University, Hospital of Nanchang University, No. 461 Bayi Ave, Nanchang, 330006 Jiangxi China

**Keywords:** Anti-infection, Cardiovascular disease, COVID-19, Marine natural products, SARS-CoV-2

## Abstract

Marine natural products (MNPs) and marine organisms include sea urchin, sea squirts or ascidians, sea cucumbers, sea snake, sponge, soft coral, marine algae, and microalgae. As vital biomedical resources for the discovery of marine drugs, bioactive molecules, and agents, these MNPs have bioactive potentials of antioxidant, anti-infection, anti-inflammatory, anticoagulant, anti-diabetic effects, cancer treatment, and improvement of human immunity. This article reviews the role of MNPs on anti-infection of coronavirus, SARS-CoV-2 and its major variants (such as Delta and Omicron) as well as tuberculosis, H. Pylori, and HIV infection, and as promising biomedical resources for infection related cardiovascular disease (irCVD), diabetes, and cancer. The anti-inflammatory mechanisms of current MNPs against SARS-CoV-2 infection are also discussed. Since the use of other chemical agents for COVID-19 treatment are associated with some adverse effects in cardiovascular system, MNPs have more therapeutic advantages. Herein, it’s time to protect this ecosystem for better sustainable development in the new era of ocean economy. As huge, novel and promising biomedical resources for anti-infection of SARS-CoV-2 and irCVD, the novel potential mechanisms of MNPs may be through multiple targets and pathways regulating human immunity and inhibiting inflammation. In conclusion, MNPs are worthy of translational research for further clinical application.

**Graphical Abstract:**

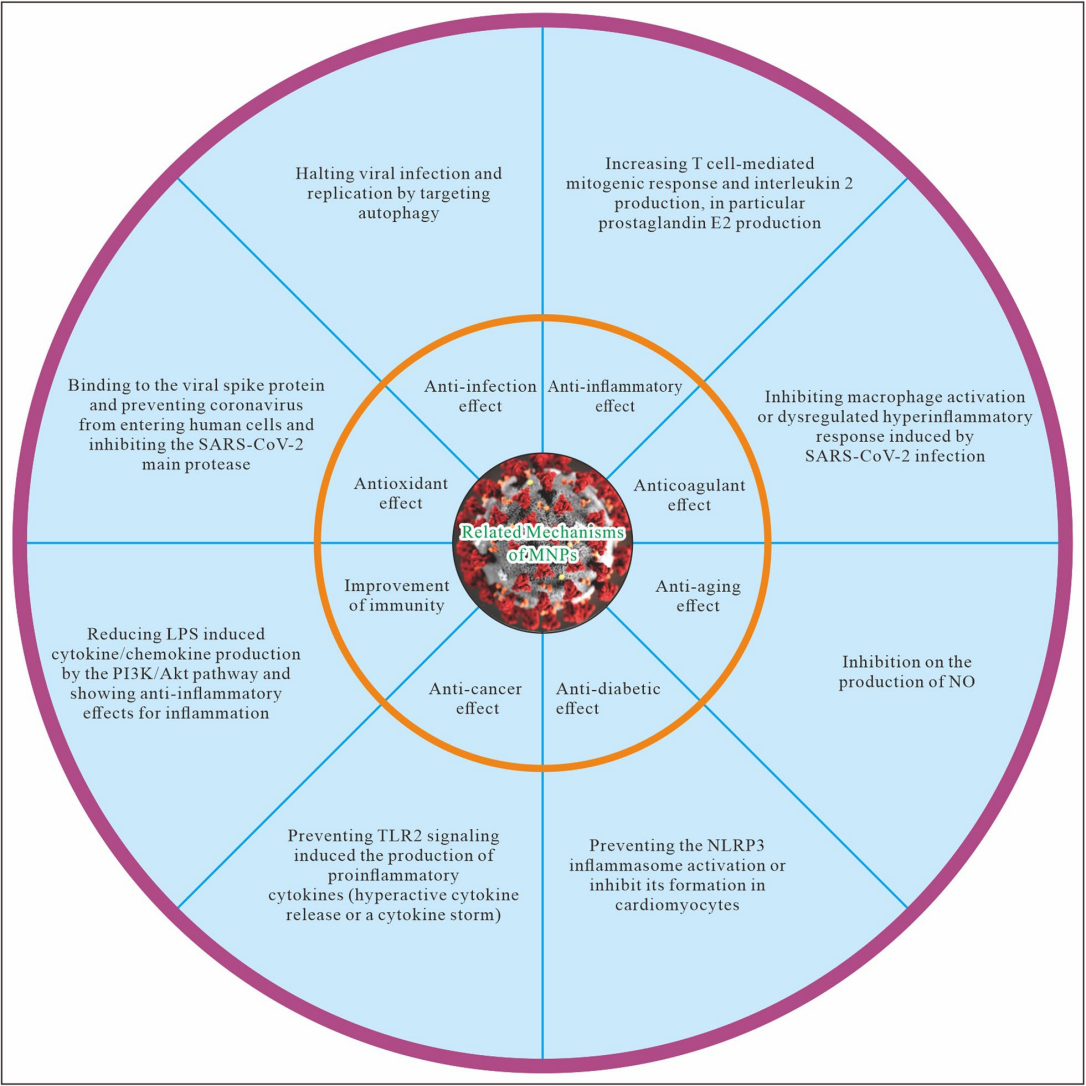

## Introduction

A comprehensive survey of deep coral reefs in the high seas showed that policymakers should give more attention to protection of ocean ecosystems and marine mammals [1, 2]. Due to a linkage with sources of sustainable food, energy, materials, biomedicine, and many others, it should be a priority attention from the United Nations for the future of this global ecosystem [3]. Currently, ocean economy is accelerating development in the globe and has become a vital support for human sustainable development. As important biomedical resources, drugs from marine organisms have long been used and exhibit unique advantages in clinical practices. Hence, it is a great task to protect and develop ocean resources and prevent and control ocean pollution since some marine compounds are also valuable tools in biomedicine and clinical applications [4, 5].

Major marine natural products (MNPs) and organisms include sea urchin, sea squirts or ascidians, sea cucumbers, sea snake, sponge, soft coral, marine algae, and microalgae. As vital biomedical resources for the discovery of marine drugs, bioactive molecules, and agents for treatment of infectious diseases and major non-communicable diseases (mNCDs), MNPs have many bioactive potentials. This article discusses the studies of MNPs as huge, novel, and promising biomedical resources for anti-infection of coronavirus (SARS-CoV-2 and its variants) and related cardiovascular disease (irCVD) as well as possible mechanisms linked to human immunity.

## MNPs: huge biomedical resources for anti-infection

As biomedical scientists, we know that marine products are beneficial to human health. The scientists are developing chemicals and novel therapeutic drugs from MNPs with anti-tuberculosis activity and H. pylori infection [6, 7], and defensive effects against viral infection, including the SARS-CoV-2 and HIV-1 [8, 9]. We expect that these compounds could be employed to treat and prevent infectious diseases (Table 1), including COVID-19 and acquired immunodeficiency syndrome (AIDS), if truly having significant antiviral activities. However, there are still huge challenges in the discovery and development of marine drugs.

**Table 1:** Marine natural products (MNPs) or marine organisms for infectious diseases and major non-communicable diseases (mNCDs)

MNPs	IDs Tuberculosis	IDs *H. Pylori* (Ulcer)	IDs HIV infection (AIDS)	IDs SARS-CoV-2 (COVID-19)	Infection-related CVD (irCVD)	mNCDs Diabetes	mNCDs Cancer & Metastasis	mNCDs Others	Mechanisms
Sea Urchin	Possible	Yes, antiulcer	Possible	Possible	Possible	Possible	Possible	Possible	Antiulcer & Healing effects
Sea Squirt (Ascidians)	Possible	Possible	Possible	Possible	Possible	Possible	Possible	Possible	Anticoagulant Activity & Anti-inflammatory Activity
Sea Cucumber	Possible	Possible	Possible	Possible	Possible	Possible	Yes	Possible	Anticoagulant Activity & Anti-inflammatory Activity
Sea Snake	Possible (Sepsis)	Possible (Sepsis)	Possible (Sepsis)	Possible (Sepsis)	Possible	Possible	Possible	Possible	Antimicrobial &Anti-inflammatory Activity
Sponge	Possible (Adjuvant Arthritis, Psoriatic Skin)	Possible (Adjuvant Arthritis, Psoriatic Skin)	Possible (Adjuvant Arthritis, Psoriatic Skin)	Possible (Adjuvant Arthritis, Psoriatic Skin)	Possible	Possible	Yes	Possible	Topical Anti-inflammatory Activity Inhibition of TNF-alpha generation & NF-kappa B Activation
Marine (red) Algae	Possible	Possible	Possible	Yes	Possible	Possible	Yes	Possible	Antiviral Activity (RNA virus)
Microalgae	Possible	Possible	Possible	Possible	Possible	Possible	Possible	Possible	Agonist of PPAR-γ Inhibit NFκB Signaling Pathway Activation
Marine organisms (vertebrate, invertebrate, Seaweed, soft coral, sea microorganism)	Possible	Possible	Possible	Yes	Yes	Possible	Possible	Possible	ACE inhibition & Anti-hypertensive Activities
Mineral-balanced deep Sea Water (MB-DSW) [Mg: Ca = 3:1]	Possible	Possible	Possible	Possible	Yes	Yes	Yes (Skin Cancer)	Possible	Anti-atopic Dermatitis Activity, Anti-diabetic, Anti-obesity Action, Cholesterol Metabolism, Activation of Autophagic Cell Death

MNPs or marine organisms are promising biomedical resources. Marine drugs, bioactive molecules, and agents can be used for treatment of infectious diseases (IDs), infection-related cardivoascular diseases (irCVD), and other major non-communicable diseases (mNCDs). They have bioactive potentials of antioxidant, anti-infection, anti-inflammatory, anticoagulant activity, anti-diabetic and anti-cancer effects

The crude extracts from marine organisms contain compounds capable of inhibiting inflammation and potential bioactive molecules [10]. Echinochrome pigment extracted from sea urchin has an insightful antiulcer healing effect [11]. Bis (3-bromo-4,5-dihydroxybenzyl) ether (C_14_H_12_Br_2_O_5_), a novel bromophenol isolated from the red alga Polysiphonia morrowii [12], is useful for treating inflammatory diseases due to the inhibition of LPS-induced inflammation in macrophage cells by inhibiting the ROS-mediated ERK signaling pathway and reducing inflammatory mediators.

As we known, MNPs are important biomedical resources for anti-infection. There are more than 1600 new steroidal structures isolated from marine organisms. Some steroids can regulate the farnesoid X receptor and the pregnane X receptor. Their novel agonists and antagonists can target human diseases, e.g., intestinal inflammation [13]. Marine invertebrate glycans (Sea squirts or ascidians and sea cucumbers) could be used as starting material for new therapeutics due to anticoagulant activity and anti-inflammation [14].

Sea cucumbers are widely consumed in traditional medicine and food. Holothuria grisea agglutinin has demonstrated the ability to modulate the inflammatory response in models of inflammation in vivo. Moreover, it is the first marine invertebrate lectin that showed an anti-inflammatory effect [15]. Fucosylated chondroitin sulfate extracted from the sea cucumber Holothuria forskali, as an inhibitor of selectin interactions, plays vital roles in inflammation and metastasis progression [16]. Sea cucumbers-derived sterol sulfate effectively attenuated inflammation by increasing serum adiponectin and reducing pro-inflammatory cytokine release [17].

A novel cathelicidin from the sea snake Hydrophis cyanocinctus, has potent both antimicrobial and anti-inflammatory activity by inhibiting the lipopolysaccharide (LPS)-induced production of nitric oxide (NO) and pro-inflammatory cytokines, such as tumor necrosis factor-α (TNF-α), Interleukin (IL)-1beta, and IL-6, is a potent candidate for the development of peptide antibiotics [18]. A small-molecule compound isolated from marine-derived fungus, bis-N-norgliovictin, significantly inhibits LPS (ligand of TLR4)-induced TNF-α production, and exhibits potent anti-inflammatory effect both in vitro and in vivo [19]. Hence, it can be a useful therapeutic candidate for the treatment of sepsis and other inflammatory diseases.

One of MNPs, marine cyanobacterium Lyngbya majuscule has a strong concentration-dependent anti-inflammatory activity by selectively inhibition the MyD88-dependent pathway [20]. As a novel marine metabolite isolated from the sponge Fasciospongia cavernosa, Cacospongionolide B showed topical anti-inflammatory activity and reduced the inflammatory response of adjuvant arthritis [21], could be used as new anti-inflammatory agents. Four drug candidates from novel bioactive sponge [22] can be used for treatment of not only inflammation but also cancer. Avarol is a marine sesquiterpenoid hydroquinone from the sponge with anti-inflammatory and antipsoriatic properties [23], it inhibits several key biomarkers up-regulated in the inflammatory response of psoriatic skin.

As bioactive molecules with the anti-inflammatory activity, microalgae-derived Oxylipins have the therapeutic potential in inflammatory diseases [24], could act as agonist of peroxisome proliferator-activated receptor gamma (PPAR-γ) and consequently inhibit nuclear factor-kappaB (NFκB) signaling pathway activation, thus lowering the production of inflammatory markers. The marine compound didemnin B decreases the activity of the cell types implicated in liver inflammation and fibrosis in vitro [25]. Other MNPs with anti-inflammatory effects include the extract of the marine sponge A. caissara [26], the sulfated galactan of the red marine alga Gelidium crinale [27], and the first marine invertebrate lectin, that is, holothuria grisea agglutinin [15]. But whether they have also antiviral effects, particularly anti-infection of SARS-CoV-2, it needs both experiments and trials to confirm their potentials.

## MNPs: novel biomedical resources for anti-infection of SARS-COV-2

As an enveloped RNA virus, coronavirus is a major cause of human respiratory diseases. The spike glycoprotein (SGP) is known as the main target of antibodies having neutralizing potency and is also considered as an attractive target for therapeutic or vaccine development. MNPs as key and novel biomedical resources for the discovery of drugs to combat the COVID-19 pandemic (Table 1), will be more and more valuable.

Among MNPs library, 17 potential SARS-CoV-2 main protease (M^pr^) inhibitors have been identified by structure-based techniques, and one of these compounds could be bioactive [28]. Marine bacteria and fungi-derived bioactive 15 compounds showed promising potential roles against SARS-CoV-2 RNA dependent RNA polymerase and methyltransferase [29]. Some new MNPs compounds (bioactive peptides) isolated from marine organisms (such as vertebrates, invertebrates, seaweeds, or other sea microorganisms) have a role of prevention on SARS-CoV-2 infection due to potential angiotensin converting enzyme (ACE) inhibition and anti-hypertensive activities [30]. The most potent marine-derived metabolite from Red-Sea invertebrates, erylosides B [31], showed a great inhibitor activity against the SARS-CoV-2 M^pro^.

Some bioactive agents from marine polysaccharides and polysaccharide-based vaccine adjuvants were developed for the fight against SARS-CoV-2 and were used as therapeutic agents and vaccines of COVID-19 [32]. A naturally existing sulfated polysaccharide, lmbda-carrageenan, purified from marine red algae, could be a promising antiviral agent for preventing infection with several respiratory viruses since this polyanionic compound exerts antiviral activity by targeting viral attachment to cell surface receptors and preventing virus entry [33]. Novel marine sulfated polysaccharides can be developed further for prophylactic as well as therapeutic purposes due to potent anti-SARS-CoV-2 activity and affinity to the SGP [34]. As potential candidates of antiviral drug, marine sulfated polysaccharides can be used to prevent SARS-CoV-2 infection [35].

Carbohydrate-binding agents from MNPs like lectins from marine algae have shown antiviral activities against SARS-CoV-2 due to targeting of N-linked glycans of the SGP envelope of CoV, and could also serve as an attractive therapeutic approach for developing novel antivirals [36]. Marine-derived natural metabolites from the soft coral [*Nephthea* sp.] can also be developed potential SARS-CoV-2 protease inhibitors [37]. As SARS-CoV-2 M^pro^ inhibitors, five MNPs (a benzo[f]pyrano[4,3-b]chromene, notoamide I, emindole SB beta-mannoside, and two bromoindole derivatives) were the most promising marine drug-like leads [38].

Up-to-date, FDA-approved marine drugs have the potential to inhibit the biological activity of SARS-CoV-2 main protease since they can bind at its active site and displace water molecules at this site [39]. The nontoxic and non-immunogenic polyphosphate, a physiological, metabolic energy (ATP)-providing polymer, could possibly also exert a protective effect against SARS-CoV-2-cell attachment [40]. These marine drugs which are already in clinical use for cancer treatment can also be used as a potential alternative to prevent and treat infected individuals with SARS-CoV-2 and its major variants (Delta and Omicron). Hence, the MNPs and their derivatives could be a promising source of structurally diverse new anti-RNA virus therapeutics [41].

## MNPs: promising biomedical resources for irCVD

Generally speaking, chronic or acute infection highly links to CVD (hypertension, myocardial infarction, arrhythmia, heart failure, and stroke), and other mNCDs, such as diabetes, cancer, respiratory and renal diseases, as well the related cardiovascular, diabetes, and cancer (CDC) strips [42, 43]. Here, the infection-related CVD is referred to as irCVD. Since MNPs have numerous health benefits, such as antioxidant, anti-infection, anti-inflammatory, anticoagulant, anti-diabetic effects, and cancer treatment [44, 45], they are not only suitable for treatment of infectious diseases, but also for control and prevention irCVD and other mNCDs (Table 1).

In fact, MNPs derived compounds extracted from marine organisms are major sources of innovative medicine. And targeting lipid metabolism may treat related diseases [46]. Diphlorethohydroxycarmalol (DPHC) isolated from ishige okamurae (a brown algae) might be a potent inhibitor for alpha-glucosidase and alpha-amylase, which can alleviates postprandial hyperglycemia in diabetic mice [47]. Cytotoxic prodiginines isolated from marine bacteria have the antimelanoma effects by provoking cytostatic rather than cytotoxic effects, cell cycle arrest at G0/G1 phase, induction of apoptosis and DNA damage, downregulation of survivin, and decreased clonogenic capacity in survivin knockdown cells [48].

Current studies showed that there are high associations between coronavirus (the SARS-CoV-2 and its variants) infection and CVD. As we known, cardiovascular health highly links to physical activity, nutrition, human immune status, and respiratory function, coronavirus can damage cardiovascular system by targeted respiratory and immune function [49]. On the one hand, COVID-9 may result in infection-related multi-organs failure in acute severe cases and mNCDs in the recovery cases, such as respiratory diseases, irCVD, and chronic kidney disease. On the other hand, as important risk factors for mortality, mNCDs are more strongly associated with outcomes and infection death in cases with COVID-19 [50].

Due to related vascular inflammation and direct vascular endothelial injury [51], SARS-CoV-2 infection may contribute to heart failure or other cardiovascular complications and multipleorgan failure. Heart failure in cases with COVID-19 involves in the abnormal activation of multiple inflammatory pathways [52]. Many studies found that a large number of cases with severe COVID-19 are easy to suffer from thrombotic complications in the venous and arterial systems. A report of an international panel showed that confirmed or suspected cases with COVID-19 infection have a high rate of acute ischemic stroke [53].

In fact, as a central feature of cases with SARS-CoV-2 infection, cerebrovascular events (stroke, ischemia, cerebrovascular injury, cerebral hemorrhage) often meet due to complement cascade, cytokine cascades, and endotheliopathy in the cerebral vasculature [54]. Thus, during the pandemic, there is an arising need of a more positive and intense thromboprophylaxis among cases hospitalized with COVID-19 due to asymptomatic deep vein thrombosis (DVT) [55].

Due to acute cardiac injury [56], cardiac arrhythmias [57, 58], major adverse cardiocerebrovascular events (MACCE) such as acute arterial events, a hypercoagulable status [59], and high mortality rate in cases with SARS-CoV-2 infection, better strategies are necessary to fight against COVID-19 and protect cardiovascular health. Hence, effective anti-infection of SARS-CoV-2 will help to protect cardiovascular system, reduce cardiac injury and cardiac arrest, and other irCVDs.

Since MNPs have a great potential role of anti-infection of SARS-CoV-2, they will also help to prevent irCVD. Some bioactive molecules extracted from marine organisms (vertebrates, invertebrates, seaweeds, or sea microorganisms) can be used not only to prevent SARS-Cov-2 infection but also to treat hypertension due to ACE inhibitory activity [30]. As one of MNPs with anticoagulant, thrombolytic, and fibrinolytic activities [60], seaweed has potential value for clinical use due to their natural origin, safety, and low cost. However, regardless of its anti-inflammatory and immunomodulatory properties, currently, no enough evidence to support the supposed favorable effects of statin (non-MNPs) therapy on COVID-19 outcomes [61].

A study found that mineral-balanced deep sea water [magnesium (Mg):calcium (Ca) = 3:1] (MB-DSW) has anti-atopic dermatitis activity due to regression of inflammatory chemokines [62]. Other studies found that MB-DSW has anti-diabetic and anti-obesity action [63] due to the stimulatory effect on mitochondrial biogenesis and function and enriched with Mg and Ca, and the effects on cholesterol metabolism [64] due to prevention of the high glucose- or FFA/glucose-induced increase of cellular cholesterol levels, and the role of the prevention of ultraviolet light-induced skin cancer development [65] due to enhancing skin cell clearance through the activation of autophagic cell death.

In addition, recombinant photoproteins from different marine organisms as a promising analytical tool have a big role in biomedical research fields [66], such as the measurement of Ca^2+^ in different intracellular compartments of animal cells, as labels in the design and development of binding assays as well as the emerging use of bioluminescence. All in all, from anti-infection of coronavirus (the SARS-CoV-2 and its variants) to preventing irCVD, MNPs are huge biomedical resources, which is worthy of developing bio-agents.

## Mechanisms linked to human immunity and future prospects

**Fig. 1:**
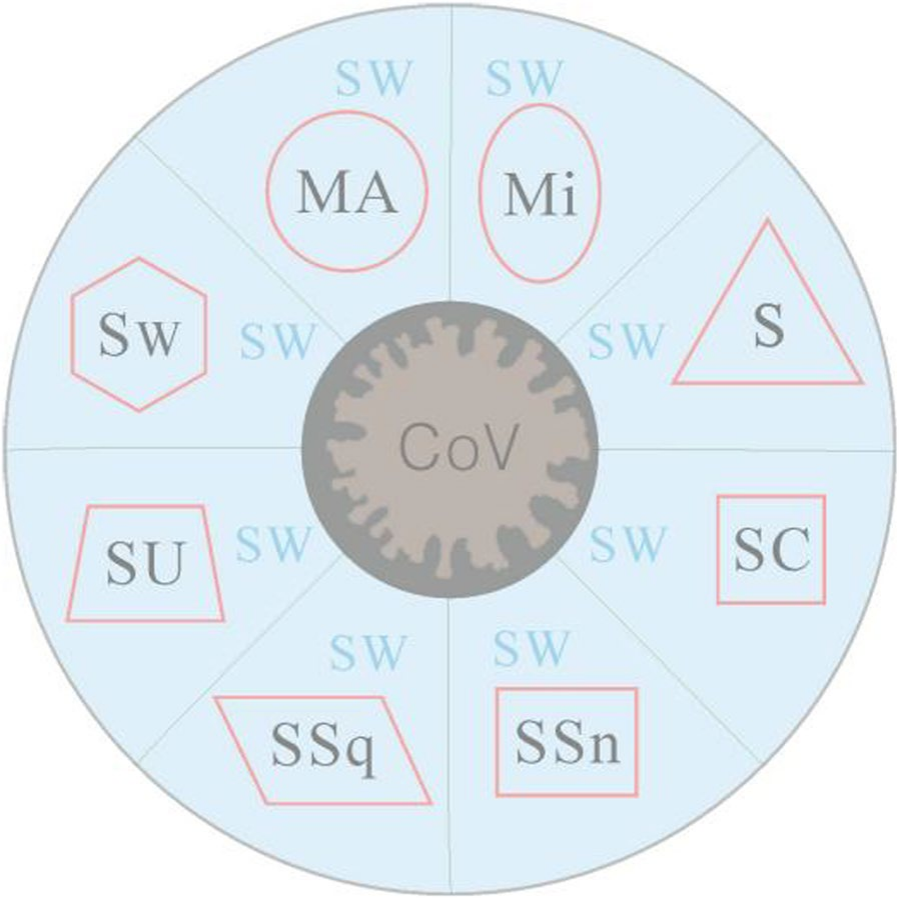
“Coronavirus (SARS-CoV-2 & Its Variants) Came, Marine Natural Products (MNPs) Halt”. Here, MA: marine (red) algae; Mi: microalgae; S: sponge; SC: sea cucumber and soft coral [*Nephthea* sp]; SSn: sea snake; SSq: sea squirt; SU: sea urchin; Sw: seaweed; SW: sea water; CoV: Coronavirus (SARS-CoV-2 & Its Variants). Whether a novel idea on “MNPs Hot Pot” will help to combat and prevent the COVID-19 pandemic, it’s worthy of doing animal experimental studies and clinical trials

Totally, this is a new era of ocean economy since biomedicine and particularly AIDS [67] and COVID-19 researches are indeed a growth industry (drug discoveries and vaccines development). The microbial flora, for example, K. pneumoniae HSL4 [68], is highly associated with industrial applications, this microbial fermentation and related biosynthesis could be also used in the field of biomedicine. Current MNPs (Fig. 1) and new marine biomedical resources as well as novel biotechnologies will help to control and combat COVID-19 infection [69] during the pandemic and post-COVID-19 era. Whatever, MNPs are worthy of developing biomedical agents for universal health coverage when combining with a magic “polypill”— healthy “environment-sleep-emotion-exercise-diet” intervention [E(e)SEEDi] lifestyle due to improvement of human immunity [70, 71].

However, marine radioactivity is a threat to human health or the environment [72]. Thus, ocean environment and marine microbes play strong roles in healthy ecosystems [73, 74]. Moreover, there are correlations between an ocean–atmosphere and human health [75], environmentally acquired infections and human disease [76, 77]. Hence, only healthy ocean & seas can meet human hope in the future. It’s time to protect ocean ecosystem for human better sustainable development.

Global food and nutrition security is very important during COVID-19 [78], especially in low- and middle-income countries [79, 80]. Currently, there is an increasing risk of both obesity and undernutrition due to the COVID-19 pandemic [81]. And due to the challenge of malnutrition (undernutrition and overnutrition) [82], for example, having suboptimal intakes of seafood [83], as food lovers [84], we should improve nutrition status with effective strategies. Since there is an association between nutrition status and COVID-19 [85], we should assess positively the nutritional risks in COVID-19 cases with useful tools [86, 87], so as to promote nutritional care and the nutrition management in these patients [88]. As a healthy diet, seafood is an important choice. New business models will improve its development [89]. And by the online-to-offline food delivery [90] during the COVID-19 pandemic, people will combat effectively the SARS-CoV-2 and its major variants (Delta, Omicron, and XBB).

Many studies have demonstrated good anti-inflammatory effects of various natural products from traditional Chinese medicine (TCM) [91]. As candidates for inhibition of SARS-CoV-2 infection, some natural products may bind to the viral spike protein and prevent it from entering human cells [92, 93]. For example, honokiol [94], and rutin [95], a medicinally important flavonoid and one of the best natural antioxidant classes, can play a role of remarkable inhibition of SARS-CoV-2 infection. Some mushroom-derived natural compounds have the potential to inhibit the SARS-CoV-2 main protease [96]. Others, like tea (*Camellia sinensis*) polyphenols [97], limonoids and triterpenoids [98], are also potential SARS-CoV-2 inhibitors. However, exception for these potentials, MNPs are also expected halting viral infection and replication by targeting autophagy, which is triggered and controlled by several signaling pathways [99].

Recent studies found that MNPs (both Brevenal and Chrysamide B) can reduce LPS induced cytokine/chemokine production and show their good performances of anti-inflammatory effects [100, 101]. The former can alter macrophage activation states and reduce inflammation in the lung, the latter has strong anti-inflammatory activity due to inhibition on the production of NO. The new potential mechanisms of MNPs against SARS-CoV-2 infection and COVID-19 may be through multiple targets and pathways regulating immunity and inhibiting inflammation. As innate immune cells, macrophage activation or dysregulated plays an important role in the hyperinflammatory response induced by SARS-CoV-2 infection [102].

As we all known, there are often cardiovascular-related conditions among patients with COVID-19 [103], such as myocarditis, acute myocardial infarction (AMI), and heart failure, that is, irCVD. Since the NACHT, leucine-rich repeat, and pyrin domain-containing protein 3 (NLRP3) inflammasome is responsible for the inflammatory response to injury or infection [104–106], whether MNPs can prevent the NLRP3 inflammasome activation or inhibit its formation in cardiomyocytes or not, it needs to further study. As we known, MNPs may not only lead to improvement in cancer induced complications but also reduce LPS-induced inflammation by the PI3K/Akt pathway due to anti-cancer and anti-inflammatory effects [107]. Since SARS-CoV-2 is prone to mutation as an RNA virus and its variants may gain resistance to available drugs or vaccines [108], “MNPs Hot Pot” as a new cocktail therapy may reduce the chances of drug resistance due to multipathways and targets so as to better protect human cardiovascular system.

As huge, novel, and promising biomedical resources, MNPs are highly expected becoming effective antiviral agents [109]. On the one hand, with the further understanding the pathogenesis of COVID-19 and the molecular mechanisms of SARS-CoV-2 infection and its variants, which involve in TLR2 signaling induced the production of proinflammatory cytokines (hyperactive cytokine release or a cytokine storm) [110], risk stratification of mild, moderate, severe COVID-19 for the acute and long-term adverse consequences [111], and human immunity [112, 113] and genetic mechanisms of critical illness [114], we can choose better clinical strategies by valuable models [115] to combat this severe viral disease.

On the other hand, due to the further understanding innate immunity and systems vaccinology [116], novel concepts and theories will help the vaccine development in this new platform and drug discoveries from current MNPs. For example, recent mRNA vaccines can effectively protect subjects from infectious disease including SARS-CoV-2 infection [117], it’s believed that we will combat finally the COVID-19 infection in the globe. Of course, just like convalescent plasma [118], large-scale clinical trials are needed to confirm the effects of these MNPs on fighting against COVID-19 and irCVD. In addition, better knowledge, attitudes, and practices [119] on the pandemic, such as incubation period [120], in-hospital mortality associated with T2D [121], vaccination effectiveness, and seasonal variations in incidence [122], are very helpful.

Currently, there are increasing threats from SARS-CoV-2 variants [123]. With more understanding of epidemiological characteristics and pathogenicity of SARS-CoV-2 variants [124, 125], human immune responses [126–128] and neutralizing antibody response [129–131] during infection and vaccinations [132], and better rapid test [133] and precise diagnosis [134] of related variants, we can combat these variants by various vaccines [135] and bispecific antibodies [136], and human immune memory, B-cells [137–139] and T cell immunity [140] by mRNA vaccines [141] or anti-Omicron antibody [142] will help to protect from infection of SARS-CoV-2 variants, and we can also predict future variants [143].

At the same time, machine learning-based models [144] are also very helpful to control the pandemic in the globe. Moreover, since the use of other chemical agents [145, 146] for COVID-19 treatment are associated with some adverse effects in cardiovascular system, MNPs have more therapeutic advantages. All in all, MNPs combined with these effective strategies [147–149], such as development of a globally scalable diagnostic biomarkers and effective antiviral targets, discovery of specific protease inhibitors or other agents, such as Paxlovid [150, 151], Molnupiravir [152], a combination of BRII-196/BRII-198 [153], as well as healthy E(e)SEEDi lifestyle [70, 71] and better nutrient strategies [154–158], will help to combat the infection of SARS-CoV-2 and its major variants (Delta, Omicron, and XBB) [159–161], thus, combat finally the COVID-19 infection.

Previous studies found that high intakes of eicosapentaenoic acid (EPA) and docosahexaenoic acid (DHA) (typically in the range of 3–5 g/d) have favorable effects on several risk factors of CVD, arterial aging and cardiovascular mortality [162], since marine-derived n-3 PUFAs increased T cell-mediated mitogenic response and interleukin 2 production, in particular prostaglandin E2 production [163]. In fact, the evidence from both epidemiologic studies and clinical trials showed both the marine-derived long-chain n-3 fatty acids EPA and DHA, and plant-derived α-Linolenic acid (ALA) [also an n-3 (ω-3) fatty acid], have the beneficial health effects for CVD (coronary heart disease, myocardial infarction, fatal cardiac arrhythmias, cardiac death, and stroke) and T2D [164, 165].

A recent study in *Science* found that taurine plays a pivotal role in anti-ageing [166]. Since marine n-3 PUFA is helpful in reducing the risk of adverse outcomes of COVID-19 infection due to potential benefits for improving human immunity [167, 168], and low marine n-3 fatty acids highly link to cardiometabolic diseases and deaths [169, 170], herein, together with “traditional Chinese medicine (TCM) Hot Pot” consisting of “Bark-Flower-Fruit-Grass-Leaf-Nucleolus(seed)-Root”(BFFGLNR) [171], MNPs with EPA, DHA, and taurine are helpful in improvement of human health as well as the recovery of COVID-19 infection by multiple targets and pathways regulating human immunity and inhibiting inflammation (Graphical Abstract).

In recent years, a series of literatures published in *Natural Products and Bioprospecting* showed that MNPs including Ascidians [172], brown algae [173], invertebrates [174], soft corals [175], some marine animals and creatures (e.g., salmon, shrimp, trout, krill, crayfish, microalgae Heamatococcus pluvialis, Chlorococcum, Chlorella zofingiensis) [176], and marine sponges [177] have exhibited a strong antimicrobial activity [173], with important and potentially bioactive compounds [174], for example, Astaxanthin [176], Cembranoids [175], and Terpenes [177], and have a broad range of biological activities, and present a huge potential for the development of various drugs for its remarkable bioactivities against mNCDs (such as CVD, cancer, diabetes, neurodegenerative and immune disorders, and others) due to a series of anti-aging [178], antioxidant, anti-inflammatory, anti-cancer, anti-diabetic, anti-obese, anti-viral (COVID-19 infection), neuro- & nephro-protective, and fertility-enhancing properties. Here, this article also summarized MNPs-derived bioactive compounds and/or targets, their chemical structures, and related potentials on human diseases (Table 2).

**Table 2:** MNPs-derived bioactive compounds and/or targets, chemical structures, and potentials on human diseases

MNPs	Chemical structures	MNPs-derived bioactive compounds or targets	Potentials	Human diseases
Marine algae (brown, green, red)	Brown Green Red	Algae-derived bioactive compounds: Alginic acid [Sargassum pallidum (Turn.) C. Ag., Sargassum fusiforme (Harv.) Setch.] Fucoxanthin and its derivatives (a natural antioxidant pigment of marine algae, including brown macroalgae and diatoms) Sulfolipids [sulfoquinovosylmonoacylglycerol (SQMG) and sulfoquinovosyldiacylglycerol (SQDG) derivatives]	Anti-cancer and anti-tumor activity Anticoagulant activities Antiviral effects	Protection against inflammatory, oxidative stress-related, nervous system, obesity, hepatic, diabetic, kidney, cardiac, skin, respiratory and microbial diseases
Microalgae (including Cyanobacteria)		Peptides, carbohydrates, and microalgal lipids, and rich in PUFAs (EPA、DHA)	Antibacterial Anticancer Anticoagulant activities Anti-inflammatory Antiviral	Anti-lung cancer Attenuation of tumor angiogenesis
Sponge (sessile invertebrates)		Bioactive compounds and secondary metabolites from marine sponges include bioactive peptides, and others, such as alkaloids, terpenoids, macrolides, polyketides endoperoxides and glycosphingolipids	Anti-biofilm activities Antimicrobial activities (antibacterial, anti-malarial, antifungal, and antiviral) Antitumor Neuroprotective activities	Antimalarials Against mucormycosis, e.g., (+)-curcudiol and (+)-curcuphenol for COVID-19-associated Mucormycosis Romotyrosine-derived alkaloids, (+)-aeroplysinin-1 against the Methicillin-resistant *Staphyllococcus aureus* (MRSA) pathogen and *Staphyllococcus aureus* Neurodegenerative disorders (Parkinson’s disease, PD)
Sea anemones (Cnidaria, Anthozoa, Actiniaria) (Others, sea fans, sea pens, and sea whips)	Cnidaria Sarcotrocheliol	Acid-sensing ion channel toxins (voltage-gated Na^+^ and K^+^ channels toxins) APEKTx1 Bcg-2 (8 to 18 kDa) & Bcg-3 (2 to 5 kDa) Bioactive peptide neurotoxins (anthopleurin-Q) Cysteine-rich polypeptide toxins Cytolysins Low molecular weight proteinaceous neurotoxins Toxins with Kunitz-type protease inhibitors activity Toxins with Phospholipase A2 activity	Anti-inflammatory pathways, energy homeostasis, cancer proliferation and painful diabetic neuropathy Molecular targets: voltage gated ion channels (potassium and sodium channel)	Development of novel drug for the treatment of ion channel-related diseases or channelopathies
Sea cucumber [Cucumaria frondosa, C. frondosa]	Frondosides	A wide range of bioactive compounds, mainly collagen, cerebrosides, glycosaminoglycan, chondroitin sulfate, saponins, phenols, and mucopolysaccharides. In particular, triterpene glycosides (Frondoside A and other)	Anticancer, anti-hypertensive, anti-angiogenic, anti-inflammatory, antidiabetic, anti-coagulation, antimicrobial, antioxidation, and anti- osteoclastogenic properties	Anticancer activity Functional Food
Sea soft coral [*Nephthea* sp]	Soft coral (Sarsolilide A)	Bioactive compounds: alkaloids, enzyme inhibitors, steroids, and terpenoids The target proteins: EGFR, VEGFR, and HER2 (erbB2) in cancer cell proliferation, growth, and survival	Anti-proliferative potential of the total soft coral extract Inhibition of tyrosine kinases, especially the EGFR type	Future anticancer drug discovery Selectively targeting multiple glioma metabolic regulators of glycolysis and glutaminolysis (one of the anti-glioma mechanisms of saponin 2)
Sea snake (e.g., the sea snake *Hydrophis cyanocinctus,* Yellow-bellied sea snake, Stokes’ sea snake)	LSP3	Bioactive compounds including peptide toxins, e.g., a novel nine-amino-acid peptide (hydrostatin-TL1, H-TL1) Targets: the various types of ion channels, i.e. K(+), Na(+), Cl(−) and Ca(2+), e.g., Kv channels	Anti-inflammatory activity in vitro and in vivo Inhibition of ion channels	Kv channel blockers from sea anemones, snakes, marine cone snails and spiders for specific human diseases, especially autoimmune disorders, inflammatory neuropathies and cancer Tumor necrosis factor (TNF)-α-associated inflammatory diseases
Sea squirt (Ascidian) (e.g., styela clava)	Ascidian	A bioactive macrolide (palmerolide A) from the Antarctic ascidian Synoicum adareanum	Anticancer	A chemotherapeutic agent for melanoma An anticancer therapeutic
Sea urchin	Glyptocidaridae	Arylalkoxynaphthalenes Protein-tyrosine phosphatase 1B (PTP1B) and PTP1B inhibitors Rich in unsaturated fatty acids, antioxidants, and minerals	Enhance immunity	A target for antiproliferative activity A therapeutic target for both T2DM and obesity
Seaweed (Sea grasses, Marine macroalgal)	Green seaweed bacteria Green seaweed fungi	A promising source of novel bioactive compounds: Alginate from brown seaweed Polysaccharides, phenolics, proteins, terpenes, lipids, and halogenated compounds from seaweed or marine macroalgal	Broad-spectrum antimicrobial activities, including antibacterial, antifungal, antiviral, anti-settlement, antiprotozoan, antiparasitic, and antitumor	Accelerated wound closure and reduced inflammation
Sea water	–	–	–	–

There are more than 60 marine-derived bioactive compounds and metabolites (Peptides, alkaloids, quinones, terpenes, polysaccharides, polyphenols, lipids, pigments, and mycotoxins) from marine organisms. These marine organisms include marine bacteria, fungi, mollusk, macro and microalgae, sponges, marine invertebrates, sea cucumber, sea urchins, seaweeds or sea grasses, soft corals, lichens, and starfish. These MNPs display various pharmaceutically significant bioactivities, including antibiotic, antiviral, neurodegenerative, anticancer, or anti-inflammatory properties. For example, related neuroprotective potentials and mechanisms in several PD hallmarks, reducing oxidative stress, preventing mitochondrial dysfunction, α-synuclein aggregation, and blocking inflammatory pathways through the inhibition translocation of NF-kB factor, reduction of human tumor necrosis factor α (TNF-α), and interleukin-6 (IL-6) as well as different intracellular signaling pathways related to PD development

## Conclusion

As important biomedical resources for the discovery of marine drugs, bioactive molecules, and agents for treatment of infectious diseases and mNCDs, current MNPs have bioactive potentials of antioxidant, anti-infection, anti-inflammatory, anticoagulant, anti-aging, anti-diabetic and anti-cancer effects as well as improvement of human immunity. They are not only huge and novel biomedical resources for anti-infection of the SARS-CoV-2 and its major variants (Delta, Omicron, and XBB), but also promising biomedical resources for control and prevention of irCVD. The related potential mechanisms of these MNPs may be through multiple targets and pathways regulating human immunity and inhibiting inflammation. Herein, it’s time to protect current ecosystem for better sustainable development of ocean economy and human cardiovascular health.

## Data Availability

The data that support the findings of this study are not publicly available but are available upon reasonable request from the corresponding author.

## References

[1] News at a glance. Science. 2020;369:1410–1. 10.1126/science.369.6510.1410 32943501

[2] Brunson JK, McKinnie SMK, Chekan JR (2018). Biosynthesis of the neurotoxin domoic acid in a blood-forming diatom. Science.

[3] World leaders are waking up to the ocean’s role in a healthy planet. Nature. 2020; 588:7–8. 10.1038/d41586-020-03301-5 33262494

[4] Scheuer PJ (1990). Some marine ecological phenomena: chemical basis and biomedical potential. Science.

[5] Stengel DB, Connan S (2015). Marine algae: a source of biomass for biotechnological applications. Methods Mol Biol.

[6] Luo X, Zhou X, Lin X (2017). Antituberculosis compounds from a deep-sea-derived fungus Aspergillus sp. SCSIO Ind09F01. Nat Prod Res.

[7] Besednova NN, Zaporozhets TS, Somova LM, Kuznetsova TA (2015). Review: prospects for the use of extracts and polysaccharides from marine algae to prevent and treat the diseases caused by Helicobacter pylori. Helicobacter.

[8] Song S, Peng H, Wang Q (2020). Inhibitory activities of marine sulfated polysaccharides against SARS-CoV-2. Food Funct.

[9] Izumida M, Suga K, Ishibashi F, Kubo Y (2019). The spirocyclic imine from a marine benthic dinoflagellate, portimine, is a potent anti-human immunodeficiency virus type 1 therapeutic lead compound. Mar Drugs.

[10] Krishnaveni M, Jayachandran S (2009). Inhibition of MAP kinases and down regulation of TNF-alpha, IL-beta and COX-2 genes by the crude extracts from marine bacteria. Biomed Pharmacother.

[11] Sayed DA, Soliman AM, Fahmy SR (2018). Echinochrome pigment as novel therapeutic agent against experimentally—induced gastric ulcer in rats. Biomed Pharmacother.

[12] Choi YK, Ye BR, Kim EA (2018). Bis (3-bromo-4,5-dihydroxybenzyl) ether, a novel bromophenol from the marine red alga *Polysiphonia morrowii* that suppresses LPS-induced inflammatory response by inhibiting ROS-mediated ERK signaling pathway in RAW 264.7 macrophages. Biomed Pharmacother.

[13] Fiorucci S, Distrutti E, Bifulco G, D’Auria MV, Zampella A (2012). Marine sponge steroids as nuclear receptor ligands. Trends Pharmacol Sci.

[14] Pavão MS (2014). Glycosaminoglycans analogs from marine invertebrates: structure, biological effects, and potential as new therapeutics. Front Cell Infect Microbiol.

[15] Moura Rda M, Aragão KS, de Melo AA (2013). Holothuria grisea agglutinin (HGA): the first invertebrate lectin with anti-inflammatory effects. Fundam Clin Pharmacol.

[16] Panagos CG, Thomson DS, Moss C (2014). Fucosylated chondroitin sulfates from the body wall of the sea cucumber Holothuria forskali: conformation, selectin binding, and biological activity. J Biol Chem.

[17] Zhang HJ, Chen C, Ding L (2020). Sea cucumbers-derived sterol sulfate alleviates insulin resistance and inflammation in high-fat-high-fructose diet-induced obese mice. Pharmacol Res.

[18] Wei L, Gao J, Zhang S (2015). Identification and characterization of the first cathelicidin from sea snakes with potent antimicrobial and anti-inflammatory activity and special mechanism. J Biol Chem.

[19] Song Y, Dou H, Gong W (2013). Bis-N-norgliovictin, a small-molecule compound from marine fungus, inhibits LPS-induced inflammation in macrophages and improves survival in sepsis. Eur J Pharmacol.

[20] Villa FA, Lieske K, Gerwick L (2010). Selective MyD88-dependent pathway inhibition by the cyanobacterial natural product malyngamide F acetate. Eur J Pharmacol.

[21] García Pastor P, De Rosa S, De Giulio A, Payá M, Alcaraz MJ (1999). Modulation of acute and chronic inflammatory processes by cacospongionolide B, a novel inhibitor of human synovial phospholipase A2. Br J Pharmacol.

[22] Andersen RJ (2017). Sponging off nature for new drug leads. Biochem Pharmacol.

[23] Amigó M, Payá M, De Rosa S, Terencio MC (2007). Antipsoriatic effects of avarol-3’-thiosalicylate are mediated by inhibition of TNF-alpha generation and NF-kappaB activation in mouse skin. Br J Pharmacol.

[24] Ávila-Román J, Talero E, de Los RC, García-Mauriño S, Motilva V (2018). Microalgae-derived oxylipins decrease inflammatory mediators by regulating the subcellular location of NFκB and PPAR-γ. Pharmacol Res.

[25] Wilson RB, Chen YJ, Sutherland BG (2020). The marine compound and elongation factor 1A1 inhibitor, didemnin B, provides benefit in western diet-induced non-alcoholic fatty liver disease. Pharmacol Res.

[26] Azevedo LG, Peraza GG, Lerner C, Soares A, Murcia N, Muccillo-Baisch AL (2008). Investigation of the anti-inflammatory and analgesic effects from an extract of Aplysina caissara, a marine sponge. Fundam Clin Pharmacol.

[27] de Sousa AA, Benevides NM, de Freitas PA (2013). A report of a galactan from marine alga Gelidium crinale with in vivo anti-inflammatory and antinociceptive effects. Fundam Clin Pharmacol.

[28] Gentile D, Patamia V, Scala A, Sciortino MT, Piperno A, Rescifina A (2020). Putative inhibitors of SARS-CoV-2 main protease from a library of marine natural products: a virtual screening and molecular modeling study. Mar Drugs.

[29] Zahran EM, Albohy A, Khalil A (2020). Bioactivity potential of marine natural products from scleractinia-associated microbes and in silico anti-SARS-COV-2 evaluation. Mar Drugs.

[30] Festa M, Sansone C, Brunet C (2020). Cardiovascular active peptides of marine origin with ACE inhibitory activities: potential role as anti-hypertensive drugs and in prevention of SARS-CoV-2 infection. Int J Mol Sci.

[31] Ibrahim MAA, Abdelrahman AHM, Mohamed TA (2021). In silico mining of terpenes from red-sea invertebrates for SARS-CoV-2 main protease (M(pro)) inhibitors. Molecules.

[32] Chen X, Han W, Wang G, Zhao X (2020). Application prospect of polysaccharides in the development of anti-novel coronavirus drugs and vaccines. Int J Biol Macromol.

[33] Jang Y, Shin H, Lee MK (2021). Antiviral activity of lambda-carrageenan against influenza viruses and severe acute respiratory syndrome coronavirus 2. Sci Rep.

[34] Tandon R, Sharp JS, Zhang F (2021). Effective inhibition of SARS-CoV-2 entry by heparin and enoxaparin derivatives. J Virol.

[35] Andrew M, Jayaraman G (2021). Marine sulfated polysaccharides as potential antiviral drug candidates to treat Corona Virus disease (COVID-19). Carbohydr Res.

[36] Gupta RK, Apte GR, Lokhande KB, Mishra S, Pal JK (2020). Carbohydrate-binding agents: potential of repurposing for COVID-19 therapy. Curr Protein Pept Sci.

[37] Abdelhafez OH, Fahim JR, Mustafa M (2021). Natural metabolites from the soft coral Nephthea sp. as potential SARS-CoV-2 main protease inhibitors. Nat Prod Res.

[38] Gaudêncio SP, Pereira F (2020). A computer-aided drug design approach to predict marine drug-like leads for SARS-CoV-2 main protease inhibition. Mar Drugs.

[39] Kalhotra P, Chittepu VCSR, Osorio-Revilla G, Gallardo-Velazquez T (2021). Field-template, QSAR, ensemble molecular docking, and 3D-RISM solvation studies expose potential of FDA-approved marine drugs as SARS-CoVID-2 main protease inhibitors. Molecules.

[40] Müller WEG, Neufurth M, Wang S, Tan R, Schröder HC, Wang X (2020). Morphogenetic (Mucin Expression) as well as potential anti-corona viral activity of the marine secondary metabolite polyphosphate on A549 cells. Mar Drugs.

[41] Christy MP, Uekusa Y, Gerwick L, Gerwick WH (2021). Natural products with potential to treat RNA virus pathogens including SARS-CoV-2. J Nat Prod.

[42] Hu CS, Tkebuchava T (2017). SEEDi1.0–3.0 strategies for major noncommunicable diseases in China. J Integr Med.

[43] Hu CS, Wu QH, Hu DY (2014). Cardiovascular, diabetes, and cancer strips: evidences, mechanisms, and classifications. J Thorac Dis.

[44] Kang HK, Seo CH, Park Y (2015). The effects of marine carbohydrates and glycosylated compounds on human health. Int J Mol Sci.

[45] Wang HD, Li XC, Lee DJ, Chang JS (2017). Potential biomedical applications of marine algae. Bioresour Technol.

[46] Cheng C, Li Z, Zhao X (2020). Natural alkaloid and polyphenol compounds targeting lipid metabolism: treatment implications in metabolic diseases. Eur J Pharmacol.

[47] Heo SJ, Hwang JY, Choi JI, Han JS, Kim HJ, Jeon YJ (2009). Diphlorethohydroxycarmalol isolated from Ishige okamurae, a brown algae, a potent alpha-glucosidase and alpha-amylase inhibitor, alleviates postprandial hyperglycemia in diabetic mice. Eur J Pharmacol.

[48] Branco PC, Pontes CA, Rezende-Teixeira P (2020). Survivin modulation in the antimelanoma activity of prodiginines. Eur J Pharmacol.

[49] Scudiero O, Lombardo B, Brancaccio M (2021). Exercise, immune system, nutrition, respiratory and cardiovascular diseases during COVID-19: a complex combination. Int J Environ Res Public Health.

[50] Drozd M, Pujades-Rodriguez M, Lillie PJ (2021). Non-communicable disease, sociodemographic factors, and risk of death from infection: a UK Biobank observational cohort study. Lancet Infect Dis.

[51] Liu F, Han K, Blair R (2021). SARS-CoV-2 infects endothelial cells in vivo and in vitro. Front Cell Infect Microbiol.

[52] Chen XM, Cao F, Zhang HM (2020). Exploration of omics mechanism and drug prediction of coronavirus-induced heart failure based on clinical bioinformatics. Zhonghua Xin Xue Guan Bing Za Zhi.

[53] Qureshi AI, Abd-Allah F, Al-Senani F (2020). Management of acute ischemic stroke in patients with COVID-19 infection: report of an international panel. Int J Stroke.

[54] Kakarla V, Kaneko N, Nour M (2021). Pathophysiologic mechanisms of cerebral endotheliopathy and stroke due to Sars-CoV-2. J Cereb Blood Flow Metab.

[55] Giorgi-Pierfranceschi M, Paoletti O, Pan A (2020). Prevalence of asymptomatic deep vein thrombosis in patients hospitalized with SARS-CoV-2 pneumonia: a cross-sectional study. Intern Emerg Med.

[56] Wang Y, Roever L, Tse G, Liu T (2020). 2019-novel coronavirus-related acute cardiac injury cannot be ignored. Curr Atheroscler Rep.

[57] Lakkireddy DR, Chung MK, Gopinathannair R (2020). Guidance for Cardiac Electrophysiology During the COVID-19 Pandemic from the Heart Rhythm Society COVID-19 Task Force; Electrophysiology Section of the American College of Cardiology; and the Electrocardiography and Arrhythmias Committee of the Council on Clinical Cardiology, American Heart Association. Circulation.

[58] Lakkireddy DR, Chung MK, Gopinathannair R (2020). Guidance for cardiac electrophysiology during the COVID-19 pandemic from the Heart Rhythm Society COVID-19 Task Force; Electrophysiology Section of the American College of Cardiology; and the Electrocardiography and Arrhythmias Committee of the Council on Clinical Cardiology, American Heart Association. Heart Rhythm.

[59] Bellosta R, Pegorer MA, Bettari L (2021). Major cardiovascular events in patients with Coronavirus Disease 2019: experience of a cardiovascular department of Northern Italy. Thromb Res.

[60] Kuznetsova TA, Andryukov BG, Makarenkova ID (2021). The potency of seaweed sulfated polysaccharides for the correction of hemostasis disorders in COVID-19. Molecules.

[61] Mitacchione G, Schiavone M, Curnis A (2021). Impact of prior statin use on clinical outcomes in COVID-19 patients: data from tertiary referral hospitals during COVID-19 pandemic in Italy. J Clin Lipidol.

[62] Lee KS, Chun SY, Lee MG, Kim S, Jang TJ, Nam KS (2018). The prevention of TNF-alpha/IFN-gamma mixture-induced inflammation in human keratinocyte and atopic dermatitis-like skin lesions in Nc/Nga mice by mineral-balanced deep sea water. Biomed Pharmacother.

[63] Ha BG, Moon DS, Kim HJ, Shon YH (2016). Magnesium and calcium-enriched deep-sea water promotes mitochondrial biogenesis by AMPK-activated signals pathway in 3T3-L1 preadipocytes. Biomed Pharmacother.

[64] Lee KS, Kwon YS, Kim S, Moon DS, Kim HJ, Nam KS (2017). Regulatory mechanism of mineral-balanced deep sea water on hypocholesterolemic effects in HepG2 hepatic cells. Biomed Pharmacother.

[65] Lee KS, Lee MG, Woo YJ, Nam KS (2019). The preventive effect of deep sea water on the development of cancerous skin cells through the induction of autophagic cell death in UVB-damaged HaCaT keratinocyte. Biomed Pharmacother.

[66] Sharifian S, Homaei A, Hemmati R, Luwor R, Khajeh K (2018). The emerging use of bioluminescence in medical research. Biomed Pharmacother.

[67] Marshall E (1996). Gallo’s institute at the last hurdle. Science.

[68] Zhou S, Li L, Perseke M, Huang Y, Wei J, Qin Q (2015). Isolation and characterization of a *Klebsiella pneumoniae* strain from mangrove sediment for efficient biosynthesis of 1,3-propanediol. Sci Bull.

[69] Huang NE, Qiao F (2020). A data driven time-dependent transmission rate for tracking an epidemic: a case study of 2019-nCoV. Sci Bull.

[70] Hu C (2018). Grants supporting research in China. Eur Heart J.

[71] Hu C (2020). Analysis of Covid-19 cases and public measures in China. SN Compr Clin Med.

[72] Smith JN, Brown RM, Williams WJ, Robert M, Nelson R, Moran SB (2015). Arrival of the Fukushima radioactivity plume in North American continental waters. Proc Natl Acad Sci U S A.

[73] Bullard EM, Torres I, Ren T, Graeve OA, Roy K (2021). Shell mineralogy of a foundational marine species, Mytilus californianus, over half a century in a changing ocean. Proc Natl Acad Sci U S A.

[74] Poff KE, Leu AO, Eppley JM, Karl DM, DeLong EF (2021). Microbial dynamics of elevated carbon flux in the open ocean’s abyss. Proc Natl Acad Sci U S A.

[75] Angle KJ, Crocker DR, Simpson RMC (2021). Acidity across the interface from the ocean surface to sea spray aerosol. Proc Natl Acad Sci U S A.

[76] Hasan NA, Grim CJ, Lipp EK (2015). Deep-sea hydrothermal vent bacteria related to human pathogenic Vibrio species. Proc Natl Acad Sci U S A.

[77] Vezzulli L, Grande C, Reid PC (2016). Climate influence on Vibrio and associated human diseases during the past half-century in the coastal North Atlantic. Proc Natl Acad Sci U S A.

[78] Carducci B, Keats EC, Ruel M (2021). Food systems, diets and nutrition in the wake of COVID-19. Nat Food.

[79] Falkendal T, Otto C, Schewe J (2021). Grain export restrictions during COVID-19 risk food insecurity in many low- and middle-income countries. Nat Food.

[80] Ali Z, Green R, Zougmoré RB (2020). Long-term impact of West African food system responses to COVID-19. Nat Food.

[81] Hawkes C, Squires CG (2021). A double-duty food systems stimulus package to build back better nutrition from COVID-19. Nat Food.

[82] Huang L, Wang Z, Wang H (2021). Nutrition transition and related health challenges over decades in China. Eur J Clin Nutr.

[83] Wang ZH, Zhai FY, Wang HJ (2015). Secular trends in meat and seafood consumption patterns among Chinese adults, 1991–2011. Eur J Clin Nutr.

[84] Nestle M (2019). A food lover’s love of nutrition science, policy, and politics. Eur J Clin Nutr.

[85] Soares MJ, Müller MJ (2020). Editorial: nutrition and COVID-19. Eur J Clin Nutr.

[86] Liu G, Zhang S, Mao Z, Wang W, Hu H (2020). Clinical significance of nutritional risk screening for older adult patients with COVID-19. Eur J Clin Nutr.

[87] Zhao X, Xu X, Li X, He X, Yang Y, Zhu S (2021). Emerging trends of technology-based dietary assessment: a perspective study. Eur J Clin Nutr.

[88] Thibault R, Coëffier M, Joly F, Bohé J, Schneider SM, Déchelotte P (2021). How the Covid-19 epidemic is challenging our practice in clinical nutrition-feedback from the field. Eur J Clin Nutr.

[89] Fletcher CA, St Clair R, Sharmina M (2021). Seafood businesses’ resilience can benefit from circular economy principles. Nat Food.

[90] Zhao X, Lin W, Cen S (2021). The online-to-offline (O2O) food delivery industry and its recent development in China. Eur J Clin Nutr.

[91] Pan MH, Chiou YS, Tsai ML, Ho CT (2011). Anti-inflammatory activity of traditional Chinese medicinal herbs. J Tradit Complement Med.

[92] Prasansuklab A, Theerasri A, Rangsinth P, Sillapachaiyaporn C, Chuchawankul S, Tencomnao T (2021). Anti-COVID-19 drug candidates: a review on potential biological activities of natural products in the management of new coronavirus infection. J Tradit Complement Med.

[93] Chen GY, Pan YC, Wu TY (2021). Potential natural products that target the SARS-CoV-2 spike protein identified by structure-based virtual screening, isothermal titration calorimetry and lentivirus particles pseudotyped (Vpp) infection assay. J Tradit Complement Med.

[94] Tanikawa T, Hayashi T, Suzuki R, Kitamura M, Inoue Y (2021). Inhibitory effect of honokiol on furin-like activity and SARS-CoV-2 infection. J Tradit Complement Med.

[95] Rahman F, Tabrez S, Ali R, Alqahtani AS, Ahmed MZ, Rub A (2021). Molecular docking analysis of rutin reveals possible inhibition of SARS-CoV-2 vital proteins. J Tradit Complement Med.

[96] Rangsinth P, Sillapachaiyaporn C, Nilkhet S, Tencomnao T, Ung AT, Chuchawankul S (2021). Mushroom-derived bioactive compounds potentially serve as the inhibitors of SARS-CoV-2 main protease: an in silico approach. J Tradit Complement Med.

[97] Singh R, Bhardwaj VK, Sharma J, Purohit R, Kumar S (2021). In-silico evaluation of bioactive compounds from tea as potential SARS-CoV-2 nonstructural protein 16 inhibitors. J Tradit Complement Med.

[98] Vardhan S, Sahoo SK (2021). Exploring the therapeutic nature of limonoids and triterpenoids against SARS-CoV-2 by targeting nsp13, nsp14, and nsp15 through molecular docking and dynamic simulations. J Tradit Complement Med.

[99] Vidoni C, Fuzimoto A, Ferraresi A, Isidoro C (2021). Targeting autophagy with natural products to prevent SARS-CoV-2 infection. J Tradit Complement Med.

[100] Keeler DM, Grandal MK, McCall JR (2019). Brevenal, a marine natural product, is anti-inflammatory and an immunomodulator of macrophage and lung epithelial cells. Mar Drugs.

[101] Zhu LQ, Fan XH, Li JF (2021). Discovery of a novel inhibitor of nitric oxide production with potential therapeutic effect on acute inflammation. Bioorg Med Chem Lett.

[102] Merad M, Martin JC (2020). Pathological inflammation in patients with COVID-19: a key role for monocytes and macrophages. Nat Rev Immunol.

[103] Dixon DL, Van Tassell BW, Vecchié A (2020). Cardiovascular considerations in treating patients with coronavirus disease 2019 (COVID-19). J Cardiovasc Pharmacol.

[104] Marchetti C, Chojnacki J, Toldo S (2014). A novel pharmacologic inhibitor of the NLRP3 inflammasome limits myocardial injury after ischemia-reperfusion in the mouse. J Cardiovasc Pharmacol.

[105] Mauro AG, Bonaventura A, Mezzaroma E, Quader M, Toldo S (2019). NLRP3 inflammasome in acute myocardial infarction. J Cardiovasc Pharmacol.

[106] Marchetti C (2019). The NLRP3 inflammasome as a pharmacological target. J Cardiovasc Pharmacol.

[107] Yang F, Cai HH, Feng XE, Li QS (2020). A novel marine halophenol derivative attenuates lipopolysaccharide-induced inflammation in RAW264.7 cells via activating phosphoinositide 3-kinase/Akt pathway. Pharmacol Rep.

[108] Singh A, Gupta V (2021). SARS-CoV-2 therapeutics: how far do we stand from a remedy?. Pharmacol Rep.

[109] Manning TJ, Thomas-Richardson J, Cowan M, Beard T (2020). Vaporization, bioactive formulations and a marine natural product: different perspectives on antivirals. Drug Discov Today.

[110] Zheng M, Karki R, Williams EP (2021). TLR2 senses the SARS-CoV-2 envelope protein to produce inflammatory cytokines. Nat Immunol.

[111] Bonaventura A, Vecchié A, Dagna L (2021). Endothelial dysfunction and immunothrombosis as key pathogenic mechanisms in COVID-19. Nat Rev Immunol.

[112] Laing AG, Lorenc A, Del Molino Del Barrio I (2020). A dynamic COVID-19 immune signature includes associations with poor prognosis. Nat Med.

[113] Ramlall V, Thangaraj PM, Meydan C (2020). Immune complement and coagulation dysfunction in adverse outcomes of SARS-CoV-2 infection. Nat Med.

[114] Pairo-Castineira E, Clohisey S, Klaric L (2021). Genetic mechanisms of critical illness in COVID-19. Nature.

[115] Han Y, Duan X, Yang L (2021). Identification of SARS-CoV-2 inhibitors using lung and colonic organoids. Nature.

[116] Pulendran B, Arunachalam PS, O’Hagan DT (2021). Emerging concepts in the science of vaccine adjuvants. Nat Rev Drug Discov.

[117] Chaudhary N, Weissman D, Whitehead KA (2021). mRNA vaccines for infectious diseases: principles, delivery and clinical translation. Nat Rev Drug Discov.

[118] Liu STH, Lin HM, Baine I (2020). Convalescent plasma treatment of severe COVID-19: a propensity score-matched control study. Nat Med.

[119] Saadatjoo S, Miri M, Hassanipour S, Ameri H, Arab-Zozani M (2021). Knowledge, attitudes, and practices of the general population about Coronavirus disease 2019 (COVID-19): a systematic review and meta-analysis with policy recommendations. Public Health.

[120] Cimolai N (2021). In pursuit of the right tail for the COVID-19 incubation period. Public Health.

[121] Kabootari M, Tirtashi RH, Hadaegh F (2022). Clinical features, risk factors and a prediction model for in-hospital mortality among diabetic patients infected with COVID-19: data from a referral centre in Iran. Public Health.

[122] Jabłońska K, Aballéa S, Toumi M (2021). The real-life impact of vaccination on COVID-19 mortality in Europe and Israel. Public Health.

[123] Layne SP, Taubenberger JK (2021). Increasing threats from SARS-CoV-2 variants: time to establish global surveillance. Sci Transl Med..

[124] Yang W, Greene SK, Peterson ER (2022). Epidemiological characteristics of the B.1.526 SARS-CoV-2 variant. Sci Adv.

[125] Munster VJ, Flagg M, Singh M (2021). Subtle differences in the pathogenicity of SARS-CoV-2 variants of concern B.1.1.7 and B.1.351 in rhesus macaques. Sci Adv.

[126] Caniels TG, Bontjer I, van der Straten K (2021). Emerging SARS-CoV-2 variants of concern evade humoral immune responses from infection and vaccination. Sci Adv.

[127] Geers D, Shamier MC, Bogers S (2021). SARS-CoV-2 variants of concern partially escape humoral but not T-cell responses in COVID-19 convalescent donors and vaccinees. Sci Immunol..

[128] Tostanoski LH, Yu J, Mercado NB (2021). Immunity elicited by natural infection or Ad26.COV2.S vaccination protects hamsters against SARS-CoV-2 variants of concern. Sci Transl Med..

[129] Zhang YN, Paynter J, Sou C (2021). Mechanism of a COVID-19 nanoparticle vaccine candidate that elicits a broadly neutralizing antibody response to SARS-CoV-2 variants. Sci Adv.

[130] Fenwick C, Turelli P, Pellaton C (2021). A high-throughput cell- and virus-free assay shows reduced neutralization of SARS-CoV-2 variants by COVID-19 convalescent plasma. Sci Transl Med.

[131] Sievers BL, Chakraborty S, Xue Y (2022). Antibodies elicited by SARS-CoV-2 infection or mRNA vaccines have reduced neutralizing activity against Beta and Omicron pseudoviruses. Sci Transl Med..

[132] Bates TA, McBride SK, Leier HC (2022). Vaccination before or after SARS-CoV-2 infection leads to robust humoral response and antibodies that effectively neutralize variants. Sci Immunol..

[133] Heggestad JT, Britton RJ, Kinnamon DS (2021). Rapid test to assess the escape of SARS-CoV-2 variants of concern. Sci Adv.

[134] de Puig H, Lee RA, Najjar D (2021). Minimally instrumented SHERLOCK (miSHERLOCK) for CRISPR-based point-of-care diagnosis of SARS-CoV-2 and emerging variants. Sci Adv.

[135] Trimpert J, Adler JM, Eschke K (2021). Live attenuated virus vaccine protects against SARS-CoV-2 variants of concern B.1.1.7 (Alpha) and B.1.351 (Beta). Sci Adv.

[136] Cho H, Gonzales-Wartz KK, Huang D (2021). Bispecific antibodies targeting distinct regions of the spike protein potently neutralize SARS-CoV-2 variants of concern. Sci Transl Med..

[137] Horiuchi S, Oishi K, Carrau L (2021). Immune memory from SARS-CoV-2 infection in hamsters provides variant-independent protection but still allows virus transmission. Sci Immunol..

[138] Kotaki R, Adachi Y, Moriyama S (2022). SARS-CoV-2 Omicron-neutralizing memory B-cells are elicited by two doses of BNT162b2 mRNA vaccine. Sci Immunol..

[139] Feldman J, Bals J, Altomare CG (2021). Naive human B cells engage the receptor binding domain of SARS-CoV-2, variants of concern, and related sarbecoviruses. Sci Immunol.

[140] Riou C, Keeton R, Moyo-Gwete T, de Oliveira T, Williamson C, Moore PL, Wilkinson RJ, Ntusi NAB, Burgers WA, South African cellular immunity network (2022). Escape from recognition of SARS-CoV-2 variant spike epitopes but overall preservation of T cell immunity. Sci Transl Med.

[141] Ying B, Whitener B, VanBlargan LA (2022). Protective activity of mRNA vaccines against ancestral and variant SARS-CoV-2 strains. Sci Transl Med..

[142] Yin W, Xu Y, Xu P (2022). Structures of the Omicron spike trimer with ACE2 and an anti-Omicron antibody. Science.

[143] Maher MC, Bartha I, Weaver S, et al. Predicting the mutational drivers of future SARS-CoV-2 variants of concern. Sci Transl Med. 2022;eabk3445. 10.1126/scitranslmed.abk3445 PMC8939770 35014856

[144] Hayawi K, Shahriar S (2022). ANTi-Vax: a novel twitter dataset for COVID-19 vaccine misinformation detection. Public Health.

[145] Mozaffari MS (2020). Role of GILZ in the kidney and the cardiovascular system: relevance to cardiorenal complications of COVID-19. J Pharmacol Exp Ther.

[146] Szendrey M, Guo J, Li W, Yang T, Zhang S (2021). COVID-19 drugs chloroquine and hydroxychloroquine, but not azithromycin and remdesivir, block hERG potassium channels. J Pharmacol Exp Ther.

[147] Fader KA, Zhang J, Menetski JP (2021). A biomarker-centric approach to drug discovery and development: lessons learned from the coronavirus disease 2019 pandemic. J Pharmacol Exp Ther.

[148] Shyr ZA, Gorshkov K, Chen CZ, Zheng W (2020). Drug discovery strategies for SARS-CoV-2. J Pharmacol Exp Ther.

[149] Zhu W, Shyr Z, Lo DC, Zheng W (2021). Viral proteases as targets for coronavirus disease 2019 drug development. J Pharmacol Exp Ther.

[150] Ledford H (2021). COVID antiviral pills: what scientists still want to know. Nature.

[151] Owen DR, Allerton CMN, Anderson AS (2021). An oral SARS-CoV-2 M(pro) inhibitor clinical candidate for the treatment of COVID-19. Science.

[152] Couzin-Frankel J (2021). Antiviral pills could change pandemic’s course. Science.

[153] ACTIV-3/Therapeutics for Inpatients with COVID-19 (TICO) Study Group. Efficacy and safety of two neutralising monoclonal antibody therapies, sotrovimab and BRII-196 plus BRII-198, for adults hospitalised with COVID-19 (TICO): a randomised controlled trial. Lancet Infect Dis. 2021;S1473-3099(21)00751-9. 10.1016/S1473-3099(21)00751-9 PMC8700279 34953520

[154] Calder PC (2021). Nutrition and immunity: lessons for COVID-19. Eur J Clin Nutr.

[155] Gregório MJ, Irving S, Teixeira D, Ferro G, Graça P, Freitas G (2021). The national food and nutrition strategy for the Portuguese COVID-19 response. Eur J Clin Nutr.

[156] Güven M, Gültekin H (2021). The effect of high-dose parenteral vitamin D_3_ on COVID-19-related inhospital mortality in critical COVID-19 patients during intensive care unit admission: an observational cohort study. Eur J Clin Nutr.

[157] Ribeiro ALR, Sousa NWA, Carvalho VO (2020). What to do when the choice is no choice at all? A critical view on nutritional recommendations for CoVID-19 quarantine. Eur J Clin Nutr.

[158] Smith ML, Sharma S, Singh TP (2021). Iodide supplementation of the anti-viral duox-lactoperoxidase activity may prevent some SARS-CoV-2 infections. Eur J Clin Nutr.

[159] Zhao H, Lu L, Peng Z (2021). SARS-CoV-2 Omicron variant shows less efficient replication and fusion activity when compared with delta variant in TMPRSS2-expressed cells. Emerg Microbes Infect..

[160] Brandal LT, MacDonald E, Veneti L (2021). Outbreak caused by the SARS-CoV-2 Omicron variant in Norway, November to December 2021. Euro Surveill.

[161] Kumar S, Thambiraja TS, Karuppanan K, Subramaniam G (2021). Omicron and Delta variant of SARS-CoV-2: a comparative computational study of spike protein. J Med Virol.

[162] Sanders TA (2014). Plant compared with marine n-3 fatty acid effects on cardiovascular risk factors and outcomes: what is the verdict?. Am J Clin Nutr.

[163] Wu D, Meydani SN, Meydani M, Hayek MG, Huth P, Nicolosi RJ (1996). Immunologic effects of marine- and plant-derived n-3 polyunsaturated fatty acids in nonhuman primates. Am J Clin Nutr.

[164] Leaf A (2008). Historical overview of n-3 fatty acids and coronary heart disease. Am J Clin Nutr.

[165] Rajaram S (2014). Health benefits of plant-derived α-linolenic acid. Am J Clin Nutr.

[166] Singh P, Gollapalli K, Mangiola S (2023). Taurine deficiency as a driver of aging. Science.

[167] Hogenkamp A, van Vlies N, Fear AL (2011). Dietary fatty acids affect the immune system in male mice sensitized to ovalbumin or vaccinated with influenza. J Nutr.

[168] Harris WS, Tintle NL, Sathyanarayanan SP, Westra J (2023). Association between blood N-3 fatty acid levels and the risk of coronavirus disease 2019 in the UK Biobank. Am J Clin Nutr.

[169] Sekikawa A, Mahajan H, Kadowaki S, Hisamatsu T, Miyagawa N, Fujiyoshi A, SESSA Research Group (2019). Association of blood levels of marine omega-3 fatty acids with coronary calcification and calcium density in Japanese men. Eur J Clin Nutr.

[170] Sun L, Zong G, Li H, Lin X (2021). Fatty acids and cardiometabolic health: a review of studies in Chinese populations. Eur J Clin Nutr.

[171] Hu C (2023). Emergency protective measures and strategies of COVID-19: from lifestyle to traditional Chinese medicine. Clin Complement Med Pharmacol.

[172] Palanisamy SK, Rajendran NM, Marino A (2017). Natural products diversity of marine ascidians (Tunicates; Ascidiacea) and successful drugs in clinical development. Nat Prod Bioprospect.

[173] Rahelivao MP, Gruner M, Andriamanantoanina H, Bauer I, Knölker HJ (2015). Brown Algae (Phaeophyceae) from the Coast of Madagascar: preliminary Bioactivity Studies and Isolation of Natural Products. Nat Prod Bioprospect.

[174] Tangerina MMP, Cesário JP, Pereira GRR, Costa TM, Valenti WC, Vilegas W (2018). Chemical profile of the sulphated saponins from the starfish luidia senegalensis collected as by-catch fauna in Brazilian coast. Nat Prod Bioprospect.

[175] Nurrachma MY, Sakaraga D, Nugraha AY, Rahmawati SI, Bayu A, Sukmarini L (2021). Cembranoids of soft corals: recent updates and their biological activities. Nat Prod Bioprospect.

[176] Patil AD, Kasabe PJ, Dandge PB (2022). Pharmaceutical and nutraceutical potential of natural bioactive pigment: astaxanthin. Nat Prod Bioprospect.

[177] Martignago CCS, Soares-Silva B, Parisi JR, Silva LCSE, Granito RN, Ribeiro AM (2023). Terpenes extracted from marine sponges with antioxidant activity: a systematic review. Nat Prod Bioprospect.

[178] Ding AJ, Zheng SQ, Huang XB, Xing TK, Wu GS, Sun HY (2017). Current perspective in the discovery of anti-aging agents from natural products. Nat Prod Bioprospect.

